# Klotho protein in neurodegenerative disorders

**DOI:** 10.1007/s10072-018-3496-x

**Published:** 2018-07-30

**Authors:** Magdalena Torbus-Paluszczak, Wojciech Bartman, Monika Adamczyk-Sowa

**Affiliations:** Department of Neurology, School of Medicine with the Division of Dentistry in Zabrze, Medical University of Silesia, Katowice, Poland, ul. 3-go Maja 13-15, 41-800 Zabrze, Poland

**Keywords:** Klotho protein, Neurodegenerative disorders, Multiple sclerosis

## Abstract

The Klotho protein is a recently discovered protein and its overexpression is associated with life extension. Klotho deficiency or silencing of the Klotho gene in mice leads to an accelerated aging and short life, whereas overexpression of Klotho in mice extends lifespan. Klotho participates in many metabolic pathways and is highly expressed in the kidneys, the choroid plexus and neurons. It plays a key role in the calcium-phosphate metabolism, remyelination, and cognitive processes. The present paper is a short review of the literature on the role of Klotho in neurodegenerative disorders, with special attention paid to multiple sclerosis. The neuroprotective function of Klotho is also reported. It is also important to consider potential clinical applications of Klotho that might be useful in the treatment of many diseases.

The Klotho protein was discovered in 1997 [1]. Its name was derived from Greek mythology. According to the beliefs, Clotho, one of three Fates, the daughter of Zeus and Themis, spun the thread of life. The other Fates were Lachesis and Atropos. The former determined the length of the thread of life, while the latter cut this thread [1, 2]. The Klotho protein was initially discovered during the production of transgenic mice that overexpress the rabbit type 1 sodium-proton exchanger. The mutation in the gene encoding Klotho in mice showed accelerated aging and shortened lifespan of mice. An insertional mutation that disrupts promoter region of the Klotho gene resulted in hypomorphic allele [1]. Four-week-old transgenic mice showed the first signs of aging, organ atrophy, atherosclerosis, vascular calcification, pulmonary emphysema, gonadal dysplasia, infertility, skin atrophy, hearing impairment, motor neuronal degeneration, thymic atrophy, osteopenia, sarcopenia, hypoglycemia, and hyperphosphatemia. These mice died prematurely at 8–9 weeks of age [1, 3, 4]. On the other hand, female mice with overexpression of the Klotho gene survived 19% longer and male mice 31% longer compared to “wild-type” mice [4]. As a hormone, Klotho binds to membrane receptors and represses intracellular signals of insulin-like growth factor 1 (IGF-1). The ability of Klotho to inhibit IGF-1 is one of the life-extending mechanisms [5].

## Structure, expression, and function of Klotho

Both in mice and humans, the Klotho gene is located on chromosome 13q12; it encompasses 50 kb and consists of 5 exons. As in the mouse gene, in the human Klotho, two transcripts encoding membrane or secretory protein are formed from one gene by alternative RNA splicing [6]. The Klotho gene encodes type 1 single-pass transmembrane glycoprotein (consisting of 1014 amino acids in mice and 1012 amino acids in humans) which is located in the cell membrane [7] and in the Golgi apparatus [8]. The intracellular domain is very short (~ 10 amino acids) without functional domains. The extracellular domain has two internal repeats, i.e., KL1 and KL2, which have an amino acid sequence homologous to family 1 glycosidases which hydrolyze beta-glycosidic linkage of saccharides, glycoproteins, and glycolipids [9]. The extracellular Klotho domain can be cleaved by membrane proteases (ADAM 10 and 17) and secreted into blood, urine, or the cerebrospinal fluid (CSF) [10, 11]. Cleaved extracellular Klotho domains act as endocrine, paracrine, or autocrine hormones on the target cells [1, 5, 8].

Three types of the Klotho protein are distinguished, i.e., cell membrane-related, intracellular, and secretory forms. Klotho associated with the cell membrane (transmembrane Klotho) can be detected in the kidneys, pituitary gland, inner ear, brain, parathyroid glands, pancreas, large intestine, skeletal muscles, bladder, ovaries, testes, and epithelial breast cells [12]. The largest concentrations are detected in distal convoluted tubules, in the kidney, and in choroid plexus of the brain [1]. Klotho is involved in the renal metabolism of calcium, phosphates, and vitamin D. The membrane Klotho forms a complex with the fibroblast growth factor receptor (FGFR) and provides selective binding affinity to the fibroblast growth factor (FGF) [13]. This complex inhibits phosphate resorption in the proximal tubule of the kidney. In the distal tubule, it also regulates Ca^2+^ absorption by stabilizing the Ca^2+^ transient receptor potential vanilloid 5 (TRPV5) channel in the plasma membrane. It inhibits renal 1-alpha 25 hydroxylase activity, thus decreasing the levels of circulating calcitriol. Therefore, hyperphosphatemia, hypercalcemia, elevated plasma calcitriol, vascular calcification, and premature aging can be observed in Klotho-deficient mice [14]. FGF23 leads to the activation of extracellular signal-regulated kinase 1 and 2 (ERK1/2), which activates serum and glucocorticoid-induced kinase 1 (SGK1) in cortical renal tubular cells. SGK1 activates with-no-lysine kinase 4 (WNK 4), stimulating WNK4-TRPV5 complex formation [15].

Klotho has been shown to inhibit 1-α hydroxylase, an enzyme responsible for the production of 1,25-dihydroxyvitamin D3, which is an active form of vitamin D [16]. Lower levels of Klotho in mice induced the formation and toxicity of vitamin D. The toxicity was reduced by limiting the formation of vitamin D [17].

On the molecular level, permanent chronic inflammation, cell proliferation disorders, or cellular aging leads to the formation of a number of age-related chronic diseases, such as obesity, diabetes, atherosclerosis, Alzheimer’s disease (AD), cancer, renal diseases, or degenerative diseases [18]. The secretory Klotho results in the reduction in TNFα and IFNγ, which can show anti-inflammatory properties. The Wnt protein is a signaling molecule that regulates intercellular interactions in the developmental period and in adult tissues. Increased signaling (activity) of Wnt disrupts the function of stem and progenitor cells and leads to cellular aging. Liu et al. [19] demonstrated that Klotho can interact with Wnt, which results in the inhibition of Wnt pathway activity, thus inhibiting the aging process. Cellular aging is also activated by oxidative stress and mitochondrial dysfunction by stimulating the p53/p21 pathways. The p53 protein is a tumor suppressor and can be activated by the ataxia telangiectasia-mutated kinase that, in turn, activates p21, which effectively inhibits cell proliferation [20]. Klotho deficiency results in the overexpression of p53/p21 by inhibiting the formation of new cells and increasing the number of aging cells [21]. Therefore, Klotho supplementation reduces cellular aging by inhibiting the p53/p21 signaling pathway [22].

## What is the role of Klotho in the central nervous system?

Klotho is produced in large amounts in the brain by ependymal cells of the choroid plexus, Purkinje EC cells, and hippocampal neurons. It is also detected in the cerebral white matter [23]. It was shown that mice lacking the Klotho gene (KL-knockout, KL-KO) showed difficulty in learning and memorizing, which occurred in early adulthood. KL-KO mice presented with a reduced number of hippocampal synapses, axonal transport disorders, and hippocampal neurodegeneration [24]. In contrast, mice overexpressing the Klotho gene (KL-OE) were characterized by significantly better cognitive functioning and better performance related to memory tasks. This effect is achieved by stimulating GluN2B subunit N-methyl-D-aspartate (NMDA) receptor [25]. Dubal et al. [25] demonstrated that Klotho nearly doubled synaptic GluN2B (through posttranscriptional mechanisms) in the hippocampus and cortex. In addition, Klotho elevation results in the increase in NMDAR-dependent genes responsible for memory consolidation, e.g., Fos. By activating NMDAR, Klotho enhances long-term potentiation (LTP), which is essential for learning and memory.

Aging of the brain is related to structural atrophy of the cortex and functional deterioration, especially in the frontal and temporal regions. The process depends on genetics. Inhibition of aging was detected in mice which were carriers of one copy of the KL-VS haplotype (composed of six single nucleotide polymorphisms, span exon 2, and its flanking sequence), which is associated with longevity [26]. Yokoyama et al. [27] demonstrated that the carriage of one KL-VS allele is associated with a larger frontal cortex (particularly right dorsolateral prefrontal cortex (rDLPFC) and the left motor area) and better executive function skill, while homozygotes showed the tendency to a smaller cortical volume and reduced functionality. Increased rDLPFC volume in KL-VS heterozygotes contributed to a better executive performance by increasing neural networks and synaptic plasticity (especially in neural networks responsible for cognitive functions). In animal models, genetic reduction of Klotho resulted in synaptic reduction [24] and demyelination [28]. Age-dependent downregulation of Klotho is also associated with an increase in methylation of GC-rich promoter. Age-related accumulation of methylation was observed in the white matter of aged rhesus monkeys. It was, however, not detected in the gray matter [29].

## Why is Klotho of particular interest for neurodegenerative diseases?

The neural Klotho can protect against the development of age-related neurodegenerative diseases. Masso et al. compared the levels of secreted Klotho (s-KL) in different brain regions between young and older mice and between triple transgenic 3xTg-AD models of AD (PS1_M146V_, APP_swe_, tau_P301L_) and healthy animals [30]. Firstly, those researchers analyzed the levels of secreted Klotho protein in the prefrontal cortex, cortex, hippocampus, and cerebellum in 6- and 18-month-old mice. The results showed that levels of the s-KL protein decreased with aging in the prefrontal cortex, cerebral cortex, and hippocampus (decrease by 41, 49, and 63%, respectively, compared to 6-month animals). The s-KL levels were similar in the cerebellum in both age groups. Expression profiles of s-KL and transmembrane Klotho (m-KL) were analyzed in the prefrontal cortex, cerebellar cortex, hippocampus, and cerebellum in 6-, 9-, and 12-month 3xTg-AD transgenic mice. As observed in 3xTg-AD mice, the expression of both transcripts decreased rapidly and occurred earlier compared to the age-matched control (significant difference at 6 months of age). Of note, at 12 months of age, s-KL and m-KL levels were similar in 3xTg-AD mice and healthy controls. Moreover, it was observed that s-KL protein levels were lower in 3xTg-AD mice compared to age-matched control. s-KL protein levels in the hippocampus were reduced by 63% in 6-month-old mice and by 53% in 12-month-old mice. In other areas of the brain, the s-KL level decreased by 31% at 12 months. The Klotho protein also plays an important role in protecting hippocampal neurons from amyloid formation and against glutamate toxicity by activating the antioxidant enzyme system. Furthermore, Klotho is necessary for the maturation of oligodendrocytes and the integrity of myelin [31]. Zeldich et al. also demonstrated a neuroprotective effect of the secreted Klotho form on hippocampal neurons exposed to the cytotoxic activity of glutamate and beta-amyloid [32].

It has recently been confirmed that Klotho is activated by the soluble amyloid precursor protein (APPsβ) [33]. Binding Klotho to APPsβ protects the central nervous system against amyloid toxicity.

## Klotho in multiple sclerosis

Multiple sclerosis (MS) is a chronic inflammatory disease that is identified as one of the major neurological disorders in young adults. The disease is characterized by the infiltration of autoreactive immune cells into the central nervous system, resulting in degeneration of the myelin and axons [34]. Costa et al. demonstrated high levels of small membrane particles (100 nm—1 μm) (known as extracellular myeloid vesicles—MVs) in MS patients. These particles emerge as significant means through which myeloid cells are capable of exerting their functions and also reflecting the activation of their progenitor cells [35]. In MS, all medications are targeted at the overactive immune response. Remyelination is another potential target for the development of new therapeutic modalities for MS [36]. During the early stages of MS, the generations of new oligodendrocytes (OLs) and myelin play an important role in the damage to the white matter. Reduced recruitment of oligodendrocyte progenitor cells (OPCs), insufficient maturation from OPCs to OLs, and a failure of OL differentiation into the mature myelin are the causes of chronic demyelinization in MS [37, 38]. Remission is mainly observed by migration of OPCs to the lesion site and their subsequent maturation into myelin-producing cells.

Zeldich et al. [36] conducted a study on the role of the Klotho protein in remyelination. In their study, they used transgenic mice with the overexpression of the Klotho transmembrane form (KL-OE), comparing it to the wild-type (WT) littermates. Demyelination was induced in mice for 6 weeks by supplying cuprizone-containing chow. Cuprizone 0.3% is a copper chelator, monoamine oxidase inhibitor, which is highly neurotoxic. At that stage of the study, rapamycin injections were also given daily to inhibit OPC proliferation. At week 7, the administration of rapamycin and cuprizone was discontinued. Remyelination was observed in both groups 3 weeks later. It was demonstrated that the number of remyelinated axons was 1.88 times higher in KL-OE mice compared to that in the wild type. In addition, the density of remyelinated axons was 1.76 times higher in KL-OE mice. The results showed a significantly beneficial effect of the Klotho protein in remyelination. That study did not demonstrate that Klotho mediated through the increased number of OPCs and OLs. However, in 2013, Chen et al. demonstrated that Klotho promoted the maturation of OPCs to OLs by ERK and Akt, leading to mTOR activation [28]. Zeldich et al. observed that the absence of the Klotho effect in this process could be explained by the fact that the mTOR pathway was blocked by rapamycin and the process could not be activated by Klotho.

Emami Aleagha et al. conducted another interesting study on the role of Klotho in MS [39]. Cerebrospinal fluid samples were collected from 22 patients with relapsing-remitting MS (RRMS) (who were not under treatment with immunomodulatory, immunosuppressant drugs or vitamin D_3_) for 6 months and 22 patients without MS (control group). In each group, the amount of the secreted form of Klotho and the “redox status” of CSF samples were investigated (using the ELISA kit and a ferric reducing antioxidant power assay—FRAP). The amount of Klotho in the CSF in RRMS was significantly lower (233.62 pg/ml) compared to that in the control group (541.62 pg/ml)—a nearly 2.3-fold decrease in comparison to the controls. The level of the FRAP in MS patients was 1.5-fold lower than in the controls—the mean for the control group was 314.93 μM, and 211.98 μM for MS patients. Additionally, a significant negative correlation was found between Klotho concentration and the Expanded Disability Status Scale (EDSS). However, a significantly positive correlation was observed between Klotho concentration and the FRAP in the CSF. Those results indicated that a decreased level of Klotho in the CSF was related to increased disability in patients with MS. Furthermore, Klotho regulates antioxidant properties of the CSF. The antioxidant power of the CSF was significantly lower in MS patients. Those results were consistent with the hypothesis according to which Klotho exerts neuroprotective effects by modulating oxidative stress.

Ahmadi et al. [40] tried to determine whether there was any change in the serum Klotho concentration in patients with MS. Forty-five patients enrolled in the study were divided into three groups. The first group consisted of 15 patients with newly diagnosed SM, with active RRMS who were not under treatment with immunosuppressants/immunomodulators or vitamin D. The second group consisted of 15 patients with active RRMS for more than 3 years (chronic group) who were under regular treatment with interferon beta-1-a. The third group included 15 non-MS patients (control group). Their results indicated that serum Klotho concentrations in new cases (585.56 pg/ml ± 153.99) were higher (but not statistically significant) compared to the control group (556.81 pg/ml ± 120.36). On the other hand, a significant increase in the serum Klotho concentration was observed in the chronic group (696.94 pg/ml ± 170.52) compared to that in the control group. No significant difference was observed between new cases and the chronic group. That study demonstrated that the serum Klotho concentration was higher in RRMS, especially in patients with long disease duration (as opposed to the above results, Klotho concentration was decreased in the CSF in patients with RRMS) [39]. It is likely that increased Klotho levels in patients with chronic MS are related to the use of immunomodulatory drugs, or it is a compensatory response to the regeneration of the nervous system and vitamin D biosynthesis. That study also confirmed the hypothesis according to which serum Klotho concentration may increase in patients with MS due to the deficiency of vitamin D (frequent abnormality in MS patients) [41]. Accordingly, transmembrane Klotho with FGF23 reduces renal vitamin D activation. The transmembrane Klotho can be cleaved and released into the bloodstream to compensate for insufficient levels of vitamin D in MS patients. Additionally, an increase in the serum Klotho concentration in the chronic group may be associated with the regeneration of nerve cells and oligodendrocytes.

## What are the potential clinical applications of Klotho?

Currently, scientists try to find a potential strategy to increase the level of Klotho in the brain. The administration of exogenous Klotho is limited by the protein weight (130 kDa)—such a large protein cannot cross the blood-brain barrier. Another way is to find molecules that act as Klotho mimetics which could enhance DNA transcription and translation and reduce Klotho degradation. Abraham et al. initiated a program to identify small molecules that could increase Klotho at the transcriptional level [17]. Two compounds were identified, i.e., compound 21 and recombinant Klotho. Both compounds induce the expression of myelin basic protein (a marker of OPC maturation) and compound 25 which enhances this effect [15]. Xuang et al. demonstrated that ligustilide, the most abundant bioactive ingredient in Rhizoma Chuanxiong (Chinese herbal medicine), upregulated Klotho expression in the cerebral choroid plexus and in serum, and inhibited IGF-1 in aged senescence-accelerated mouse prone-8 (SAMP8) mice. Treatment with ligustilide reduced memory deficits, amyloid-β (1)-42 accumulation, tau protein phosphorylation, and neuronal loss, thus demonstrating potential neuroprotective effects against AD [42]. Wang et al. investigated beneficial effects of tubastatin A (TubA), a new specific inhibitor of HDAC6 in an animal model of acute cerebral ischemia. Those researchers also demonstrated that the level of FGF21 protein (a regulator of glucose and lipid metabolism that plays a neuroprotective role against glutamate toxicity) was significantly reduced under ischemic conditions. As a cofactor of the FGF receptor, β-Klotho is essential for FGF21 signaling, and TubA significantly enhances the β-Klotho mRNA level in the ischemic cortex [43]. Schafer et al. investigated the effect of long-term calorie restriction (CR) on the CA1 hippocampal region—one of the most vulnerable areas to age-related pathologies such as AD. It was demonstrated that CR endogenously increased Klotho mRNA levels in the hippocampal CA1 region in both younger-adult and older-adult mice, thus delaying the transition from adulthood to old age [44]. The Klotho protein can also be affected by physical activity—the interaction is mediated by growth factors, particularly the FGF and IGF-1 receptors. Additionally, physical activity and Klotho protein increase resistance to oxidative stress due to the influence of Klotho on various antioxidant and stress-resistant proteins. Exercise-dependent production of growth factors such as brain-derived neurotrophic factor (BDNF) may improve neurogenesis and play a key role in maintaining a high level of cognitive processes [45].

Latanoprost (LA) is an ocular hypotensive agent used in the treatment of glaucoma targeting prostaglandin F2α (FP) receptor. The study of Yamamoto et al. demonstrated that a neuroprotective effect of LA is due to the Klotho-mediated suppression of calpain activation [46]. It was shown that LA is transported to retinal ganglion cells (RGCs) via organic anion transporting polypeptide 2B1 (OATP2B1) where it activates ADAM17 via the PKC pathway. Klotho fragments can also inhibit the influx of Ca2+ to RGCs and then induce calpain inhibition. Calpain inhibitors (and Klotho as a regulator of calpain activity) are promising therapeutic targets for the prevention of neurodegeneration.

## Summary

Klotho is highly expressed in the brain. Many studies have demonstrated a neuroprotective role of Klotho. In the human brain, the secreted isoform of the Klotho protein plays a crucial role in neuronal activity, which is the key to aging and neurodegenerative processes. Due to activation of OPCs, soluble Klotho could be used as a therapeutic strategy for brain tissue regeneration. As a cofactor that modulates affinity for various receptors, Klotho regulates calcium homeostasis and oxidative stress. In the CSF, Klotho can participate in the suppression of cytokine/chemokine expression in the central nervous system, supporting the activation of neuronal cells. From a clinical point of view, the Klotho level in the CSF can be used as a biomarker of MS severity. Although it is still not known why Klotho suppresses aging, maintaining Klotho levels by stimulating endogenous Klotho production or administering exogenous Klotho protein may be a potential therapeutic target to slow down the aging process and reduce the incidence of age-related diseases. Finally, as a regulator of remyelination in the white matter, Klotho can become a new therapeutic substance for MS and other demyelinating and neurodegenerative diseases.

## References

[1] Kuro-o M, Matsumura Aizawa Y (1997). Mutation of the mouse Klotho gene leads to a syndrome resembling ageing. Nature.

[2] Małyszko J: Białko Klotho a przewlekła choroba nerek. Forum Nefrologiczne 2009; tom 2, nr 2: 69–73

[3] Wang Y, Sun Z (2009). Current understanding of Klotho. Ageing Res Rev.

[4] Kurosu H, Kuro-o M (2009). The Klotho gene family as a regulator of endocrine fibroblast growth factors. Mol Cell Endocrinol.

[5] Kurosu H, Yaamoto M, Clark JD (2005). Suppression of aging in mice by the hormone Klotho. Science.

[6] Matsumura Y, Aizawa H, Shiraki-Iida T, Nagai R, Kuro-o M, Nebeshima Y (1998). Identification of the human klotho gene and its two transcripts encoding membrane and secreted klotho protein. Biochem Biophys Res Commun.

[7] Shiraki-Iida T, Aizawa H, Matsumura Y, Sekine S, Iida A, Anazawa H, Nagai R, Kuro-o M, Nabeshima YI (1998). Structure of the mouse klotho gene and its two transcripts encoding membrane and secreted protein. FEBS Lett.

[8] Imura A, Tsuji Y, Murata M, Maeda R, Kubota K, Iwano A, Obuse C, Togashi K, Tominaga M, Kita N, Tomiyama K (2007). Iijima et al : alpha-klotho as a regulator of calcium homeostasis. Science.

[9] Tohyama O, Imura A, Iwano A, Freund JN, Henrissat B, Fujimori T, Nabeshima YI (2004). Klotho is a novel beta-glucuronidase capable of hydrolyzing steroid beta-glucuronides. J Biol Chem.

[10] Chen CD, Podvin S, Gillespie E, Leeman SE, Abraham CR (2007). Insulin stimulates the cleavage and release of the extracellular domain of Klotho by ADAM10 and ADAM17. Proc Natl Acad Sci U S A.

[11] Bloch L, Sineshchekova O, Reichenbach D, Reiss K, Saftig P, Kuro-o M, Kaether C (2009). Klotho is a substrate for alpha-, beta- and gamma-secretase. FEBS Lett.

[12] Kameroni M, Ohyama Y, Kurabayashi M, Takahashi K, Nagai R, Furuya N (2002). Expression of Klotho protein in the inner ear. Hear Res.

[13] Kim JH, Hwang KH, Park KS, Kong ID, Cha SK (2015). Biological role of anti-aging protein Klotho. J Lifestyle Med.

[14] Drüeke TB, Prie D (2007). Klotho spins the thread of life-what does Klotho do to the receptors of fibroblast growth factor-23 (FGF23). Nephrol Dial Transplant.

[15] Andrukhova O, Smorodchenko A, Egerbacher M, Streicher C (2014). FGF23 promotes renal calcium reabsorption through the TRPV5 channel. EMBO J.

[16] Yoshida T, Fujimori T, Nabeshima Y (2002). Mediation of unusually high concentrations of 1,25-dihydroxyvitamin D in homozygous klotho mutant mice by increased expression of renal 1α-hydroxylase gene. Endocrinology.

[17] Abraham CR, Chen CD, Cuny GD, Glicksman MA, Zeldich E (2012). Small-molecule Klotho enhancers as novel treatment of neurodegeneration. Future Med Chem.

[18] Zhu Y, Armstrong JL, Tchkonia T, Kirkland JL (2014). Cellular senescence and the senescent secretory phenotype in age-related chronic disease. Curr Opin Clin Nutr Metab Care.

[19] Liu H, Ferguson MM, Castilho RM (2007). Augmented Wnt signaling in mammalian model of accelerated aging. Science.

[20] Munoz-Espin D, Serrano M (2014). Cellular senescence: from physiology to pathology. Nat Rev Mol Cell Biol.

[21] de Oliveira RM (2006). Klotho RNAi induces premature senescence of human cells via a p53/p21 dependent pathway. FEBS Lett.

[22] Ikushima M, Rakugi H, Ishikawa K, Maekawa Y, Yamamoto K, Ohta J, Chihara Y, Kida I, Ogihara T (2006). Anti-apoptotic and antisenescence effects of Klotho on vascular endothelial cells. Biochem Biophys Res Commun.

[23] Duce JA, Podvin S, Hollandar W, Kipling D, Rosene DL, Abraham CR (2008). Gene profile analysis implicates Klotho as an important contributor to aging changes in brain white matter of the rhesus money. Glia.

[24] Shiozaki M, Yoshimura K, Shibata M, Koike M, Matsuura N, Uchiyama Y, Gotow T (2008). Morphological and biochemical signs of age-related neurodegenerative changes in klotho mutant mice. Neuroscience.

[25] Dubal DB, Yokoyama JS, Zhu L, Broestl L, Worden K, Wang D, Sturm VE, Kim D, Klein E, Yu GQ, Ho K, Eilertson KE, Yu L, Kuro-o M, de Jager PL, Coppola G, Small GW, Bennett DA, Kramer JH, Abraham CR, Miller BL, Mucke L (2014). Life extension factor klotho enhances cognition. Cell Rep.

[26] Arking DE, Krebsova A, Macek M, Macek M, Arking A, Mian IS, Fried L, Hamosh A, Dey S, McIntosh I, Dietz HC (2002). Association of human aging with a functional variant of klotho. Proc Natl Acad Sci U S A.

[27] Yokoyama JS, Sturm VE, Bonham LW, Klein E, Arfanakis K (2015). Variation in longevity gene Klotho is associated with greater cortical volumes. Ann Clin Transl Neurol.

[28] Chen CD, Sloane JA, Li H (2013). The antiaging protein Klotho enhances oligodendrocyte maturation and myelination of the CNS. J Neurosci.

[29] King GD, Rosene DL, Abraham CR (2012). Promoter methylation and age-related downregulation of Klotho in rhesus monkey. Age.

[30] Masso A, Sanchez A, Gimenez-Llort L (2015). Secreted and transmembrane αKlotho isoforms have different spatio-temporal profiles in the brain during aging and Alzheimer’s disease progression. PLoS One.

[31] Abraham CR, Mullen PC, Tucker-Zhou T, Chen CD, Zeldich E (2016). Klotho is a neuroprotective and cognition-enhancing protein. Vitam Horm.

[32] Zeldich E, Chen CD, Colvin TA, Bove-Fenderson EA, Liang J, Tucker Zhou TB, Harris DA, Abraham CR (2014). The neuroprotective effect of Klotho is mediated via regulation of members of the redox system. J Biol Chem.

[33] Li H, Wang B, Wang Z, Guo Q, Tabuchi K, Hammer RE, Sudhof TC, Zheng H (2010). Soluble amyloid precursor protein (APP) regulates transthyretin and Klotho gene expression without rescuing the essential function of APP. Proc Natl Acad Sci U S A.

[34] Steinman L (2014). Immunology of relapse and remission in multiple sclerosis. Annu Rev Immunol.

[35] Dalla Costa G, Finardi A, Garzetti L, Carandini T, Comi G, Martinelli V, Furlan R (2018). Disease-modifying treatments modulate myeloid cells in multiple sclerosis patients. Neurol Sci.

[36] Zeldich E, Chen CD, Avila R, Medicetty S, Abraham CR (2015). The anti-aging protein Klotho enhances remyelination following cuprizone-induced demyelination. J Mol Neurosci.

[37] Chang A, Tourtellotte WW, Rudick R, Trapp BD (2002). Premyelinating oligodendrocytes in chronic lesions of multiple sclerosis. N Engl J Med.

[38] Chang A, Staugaitis SM, Dutta R, Batt CE, Easley KE, Chomyk AM, Yong VW, Fox RJ, Kidd GJ, Trapp BD (2012). Cortical remyelination: a new target for repair therapies in multiple sclerosis. Ann Neurol.

[39] Emami Aleagha MS, Siroos B, Ahmadi M, Balood M, Palangi A, Haghighi AN, Harirchian MH (2015). Decreased concentration of Klotho in the cerebrospinal fluid of patients with relapsing–remitting multiple sclerosis. J Neuroimmunol.

[40] Ahmadi M, Emami Aleagha MS, Harirchian MH, Yarani R, Tavakoli F, Siroos B (2016). Multiple sclerosis influences on the augmentation of serum Klotho concentration. J Neurol Sci.

[41] Nieves J, Cosman F, Herbert J, Shen V, Lindsay R (1994). High prevalence of vitamin D deficiency and reduced bone mass in multiple sclerosis. Neurology.

[42] Kuang X, Chen YS, Wang LF, Li YJ, Liu K, Zhang MX, Li LJ, Chen C, He Q, Wang Y, du JR (2014). Klotho upregulation contributes to the neuroprotection of ligustilide in an Alzheimer’s disease mouse model. Neurobiol Aging.

[43] Wang Z, Leng Y, Wang J, Liao HM, Bergman J, Leeds P, Kozikowski A, Chuang DM (2016). Tubastatin A, an HDAC6 inhibitor, alleviates stroke-induced brain infarction and functional deficits: potential roles of α-tubulin acetylation and FGF-21 up-regulation. Sci Rep.

[44] Schafer MJ, Dolgalev I, Alldred MJ, Heguy A, Ginsberg SD (2015). Calorie restriction suppresses age-dependent hippocampal transcriptional signatures. PLoS One.

[45] Foster PP, Rosenblatt KP, Kuljiš RO (2011). Exercise-induced cognitive plasticity, implications for mild cognitive impairment and Alzheimer’s disease. Front Neurol.

[46] Yamamoto K, Sato K, Yukita M et al (2016) The neuroprotective effect of latanoprost acts via klotho-mediated suppression of calpain activation after optic nerve transection. J Neurochem. 10.1111/jnc.13902:1-14. 10.1111/jnc.13902 PMC5299490 27859240

